# Undamped climate change poses the need for substantial shifts in cultivated crop types in Germany

**DOI:** 10.1038/s41598-026-42040-x

**Published:** 2026-03-02

**Authors:** Luzia Keupp, Andreas Hotho, Stefan Dech, Heiko Paeth

**Affiliations:** 1 Institute of Geography and Geology, University of Würzburg, Würzburg, Germany; 2 Center for Artificial Intelligence and Data Science, University of Würzburg, Würzburg, Germany; 3 German Remote Sensing Data Center, German Aerospace Center, Oberpfaffenhofen, Germany

**Keywords:** climate analogues, regional climate impact on agriculture, agriculturally relevant climate indices, CROPGRIDS, stakeholder involvement, multivariate climate characteristics, Climate sciences, Environmental sciences

## Abstract

Numerous studies have highlighted the severe implications of climate change for agriculture across the planet, due to shifting climate patterns, heat waves, flood and drought events. Combining state-of-the-art high-resolution observational and model-based climate data with elaborate land use data and the method of climate analogues now allows to foreshadow future agricultural landscapes, anticipating adaptational and transformational needs towards enhanced climate resilience of regional agriculture. The approach is exemplified for a highly diversified farming area in southern Germany. Until the end of the 21st century a clear shift towards typical Mediterranean crops prevails, while many of today’s common crops are expected to become less important. Only higher-elevation parts are projected to face climate analogues in Germany itself, whereas most subregions may exhibit climate conditions that are nowadays found in the northern Mediterranean area. Altogether, undamped climate change will require a radical transformation of the German agricultural sector.

**Supplementary Information:**

The online version contains supplementary material available at 10.1038/s41598-026-42040-x.

## Introduction

Agriculture is among the most sensitive economic sectors to current and future climate change^1,2^. Until the end of the 21st century, the Intergovernmental Panel on Climate Change expects increasing crop failure, especially in subtropical and tropical regions, the Mediterranean region as well as parts of central Europe, including high damage due to meteorological extremes, pests, and plant diseases leading to rising food prices and lower food quality, with mitigation failure implying substantially larger efforts in adaptation^3^. Numerous studies have reported on the challenging needs of adaptation in the agricultural sector including forestry, fishery, and specialized crops such as grapevine, fruits, and vegetables [e.g.,^4–7^]. These challenges are not confined to tropical and subtropical regions of rain-fed agriculture where crop yield and food security may indeed be most affected^8^. According to ref^9^, 98% of all agricultural production areas across Europe may experience substantially different agro-climatic conditions by the middle of our century.

The present study is dedicated to a region in southern Germany that for many decades was not associated with crop failure and adaptational requirements in the agricultural sector. However, in practically all years since 2015, up to 50% loss of crop yield were caused by severe heat waves and deficient rainfall during the growing season, partly interrupted by heavy rain events and local flooding^10^. In fact, Germany is characterized by above-average warming rates and a tendency towards persistent weather patterns that cause hot and dry episodes during summer^11,12^. Southern Germany represents a hot spot of regional climate change, resulting from a nexus of enhanced greenhouse conditions, altered circulation patterns and planetary wave activity, reduced snow cover in winter, and a relatively high level of population density and soil sealing^12,13^.

**Fig. 1:**
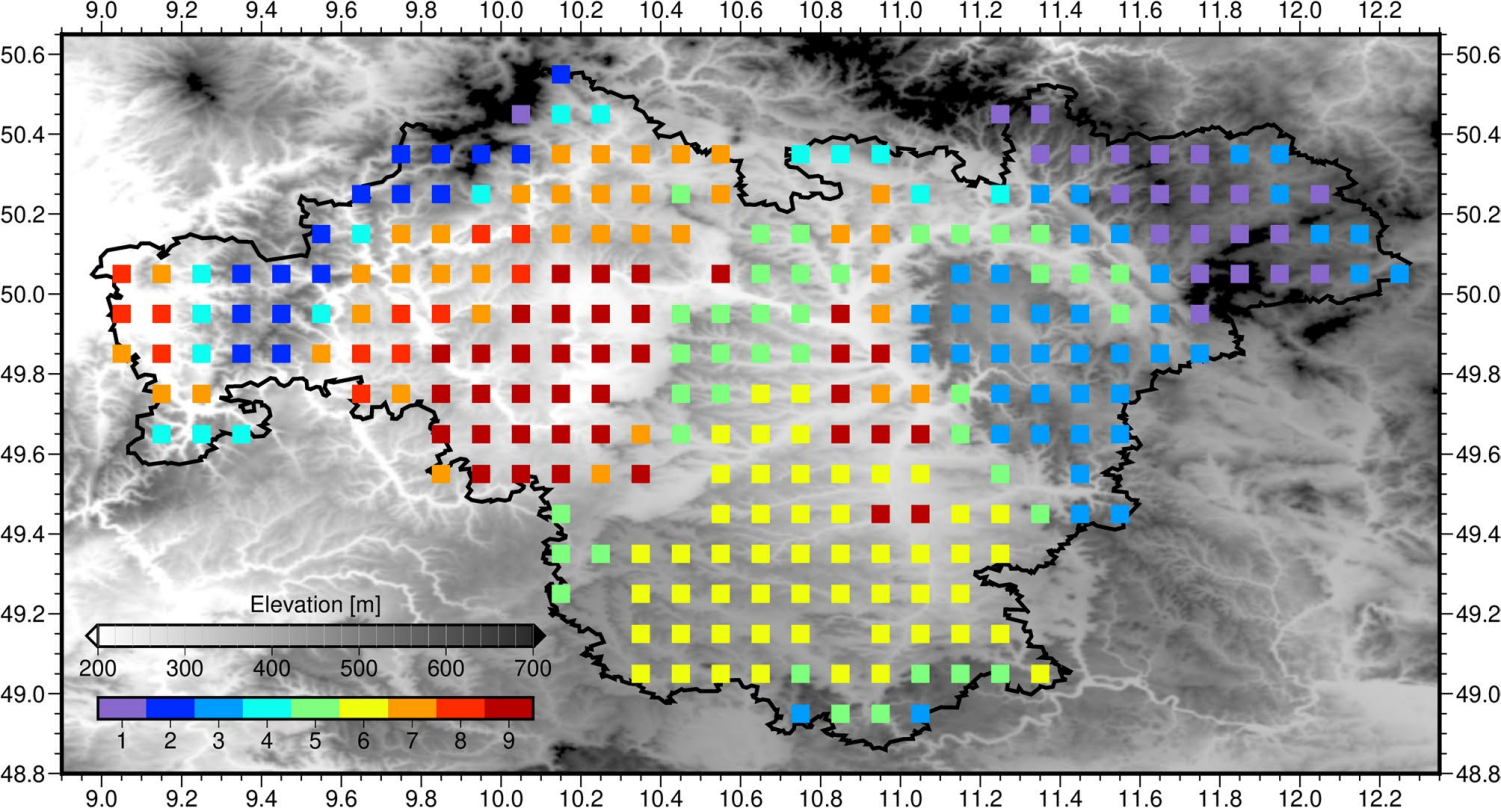
Nine clusters of Franconian climate during the 2070–2099 period, based on a multi-model ensemble of high-resolution regional climate model projections under the RCP8.5 emission scenario. The cluster numbers are ranked from relatively cold (1) to relatively warm (9). Franconia is framed. Figure created using GMT—The Generic Mapping Tools, Version 6.5.0 (https://www.generic-mapping-tools.org/). Relief shading based on SRTM15 data^26^.

The study domain covers the region of Franconia in northern Bavaria, comprising the governmental districts of Lower, Middle, and Upper Franconia (cf. Supplementary Fig. S1). The region extends over an area of about 23,000 km^2^ and is centred at around 50°N and 11°E (cf. Fig. 1). Its main characteristics are a heterogeneous topography with elevations between 100 and 1,051 m asl., diversified land use patterns and intense activity in agriculture, viticulture, forestry, fruit and vegetable growing. In total, agriculture has a 44% areal coverage in Franconia^14^, highlighting its socioeconomic importance (cf. Supplementary Fig. S1). Due to heterogeneous geological and geomorphological conditions, the soil pattern is also quite diverse and affected by human impacts since the Neolithic time^15^. Due to its location, heterogeneous landscape and diverse land-use pattern, we put the following motivational conjecture regarding our case study ahead of the actual study: the region of Franconia typifies Central Europe’s climate in a nutshell and is representative of Central European agriculture.

The central part of the study is then guided by the following scientific hypothesis: regional climate change imposes a substantial adaptational effort on German farmers since the spectrum of climate-adapted crop plants extensively changes when radiative forcing continuously increases until 2100. To address this hypothesis, our investigation pursues a different approach compared to many previous studies on the climate-agriculture nexus, where challenges for agriculture are qualitatively derived from changes in temperature, precipitation and relevant extreme events based on plausibility assumptions [e.g.,^9,10,16^]. The novel aspect of the present study relies on a sophisticated approach of climate analogues combined with very high-resolution data on present-day crop plant patterns across Europe. To gain insight into the land-use patterns under future climate conditions in Franconia, regions are identified that exhibit this future climate already today, usually because they are located equatorward of Germany. These so-called climate analogue regions have often been analysed for urban regions to identify twin cities in a climatological sense^17–19^. Most studies rely on a very limited number of variables, leading to the problem of erratic patterns of climate analogues^20–23^. The application of climate analogues to agricultural issues is still underrepresented, e.g., for winegrowing^20,23^, forestry^21,24^, or crop productivity^22^.

Our study suggests three methodical steps to address the underlying hypothesis and to make a useful general contribution to the climate analogue approach:

First, a large set of 28 climate indicators relevant to agriculture is identified from the literature and from a survey among more than 30 farms in the study domain. Based on this tailored multivariate setting the future climate of Franconia is split up via cluster analysis into nine subregional types spanning from relatively warm and dry plains to cold and humid low-mountain ridges.

Second, the climate analogue regions are found by using the absolute lower and upper limits of the future climate in combination with an Euclidian metric as a relative measure of distance. The identification of the analogue regions is further confined by information on soil characteristics, leading to pairs that closely resemble each other in terms of the natural landscape.

Third, the present-day crop spectra of the Franconian subregions are compared with the present-day crop spectra of the analogue regions using the lately published high-resolution land use dataset CROPGRIDS^25^. The latter serve as a first glimpse on the spectrum of crop plants that may be cultivated under the expected future climatic conditions in the Franconian subregions.

With this approach, we expect to provide useful information for the climate-induced transformation process in German agriculture. Farmers may want to conduct timely experiments with new climate-adapted crops, especially for perennial crops such as grapevines or fruit trees.

## Results

### Assessment and internal classification of future Franconian climate

The agroclimatic typification of the study domain is based on 28 climate indicators that have been derived from the literature and from a survey among farmers across Franconia collaborating with us in the framework of the EU-funded project BigData@Geo 2.0 (https://www.bigdata-at-geo.eu) (see Supplementary Table S1). The climate indicators are based on daily minimum, maximum, and mean temperature as well as precipitation. They describe important features of crop growing such as thermal and hygric (precipitation or moisture related) conditions, cold, warm, dry and flood events, climate seasonality and continentality, as well as vegetation period (see Supplementary Table S2). The climate analogue approach requires that the future climate of the target region is assessed. We rely on a quality-approved multi-model ensemble of regional climate model simulations from the CORDEX framework that achieved a new benchmark in horizontal resolution. Seven long-term transient simulations with a 0.11° horizontal resolution are available until the end of the 21st century (Supplementary Table S3). The RCP8.5 emission scenario is chosen to evaluate the upper limit of future transformations in Franconian agriculture for the end of the 21st century, as it implies a global warming of more than 4 °C for the end of the 21st century compared to pre-industrial conditions^2^.

Due to the multi-collinearity among such climate indicators, a principal component (PC) analysis (for 2070–2099) is carried out leading to a smaller subset of complementary variables that are more appropriate to identify subregional climate types. The leading two PCs account for almost 80% of the total variability. The first PC represents thermal and hygric conditions that are closely tied to each other due to the topographic heterogeneity of the study domain. The second PC relates to climate’s continentality and seasonality (Supplementary Fig. S2, cf. Methods).

**Fig. 2:**
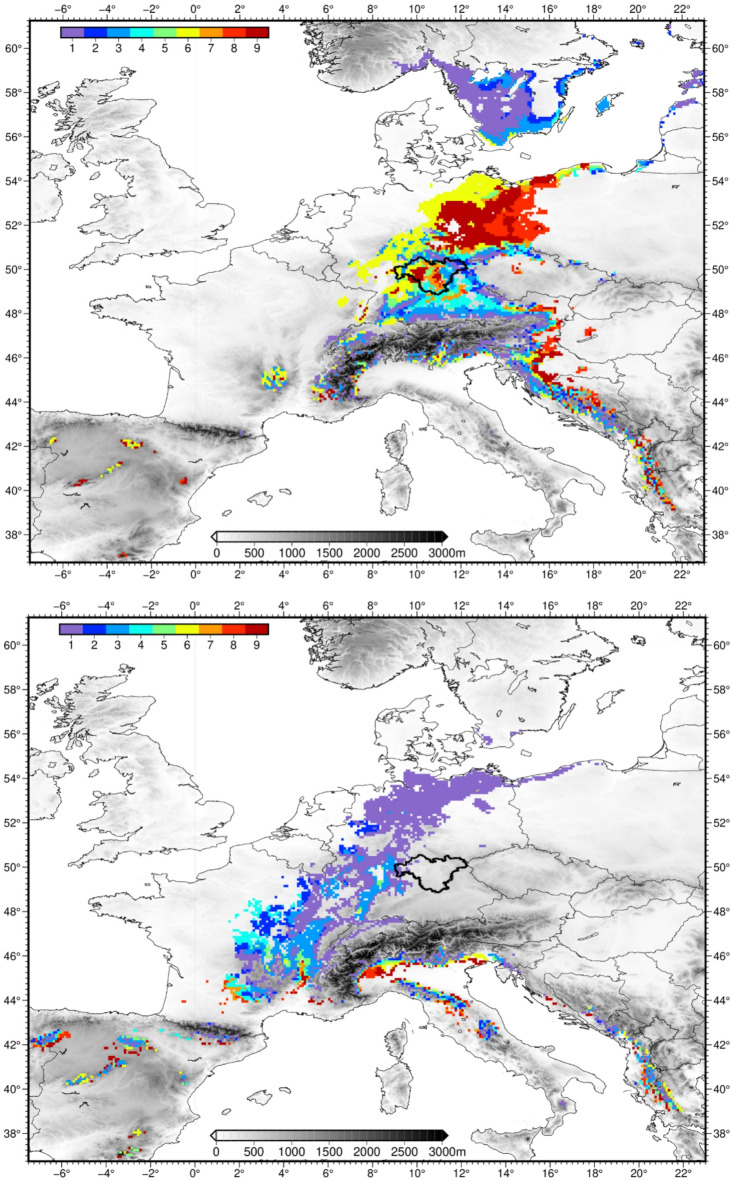
Analogue climate regions of the classified nine Franconian sub-climates (see Fig. 1) under present-day (1993–2022) climate conditions in Franconia (top panel) and under future (2070–2099) climate conditions in Franconia (bottom panel). Franconia is framed. Figure created using GMT—The Generic Mapping Tools, Version 6.5.0 (https://www.generic-mapping-tools.org/). Relief shading based on SRTM15 data^26^; shorelines from GSHHG^28^.

A cluster analysis is applied to the first and second PC to subdivide Franconia into nine subregions of homogeneous climate each (Fig. 1). The pattern primarily reflects the topographic structure of the study region (which is also depicted in Fig. 1) with colder and wetter climate extending over low maintain ranges, whereas warmer and dryer conditions prevail in the plains in between and along river valleys. The associated mean climate characteristics listed in Table 1 span from a colder, more humid high-elevation climate type with shorter vegetation period in cluster 1 to a warmer and dryer climate in the valleys and plains with longer dry spells and vegetation period in cluster 9. When considering the quantitative differences among the clusters, Franconia can be regarded as “Central European climate in a nutshell”, except for the maritime northwestern parts. This will later be demonstrated by a present-day climate analogues analysis (cf. Fig. 2, top). The numbers in brackets reveal the mean changes during the growing season with respect to the reference climate at the end of the 20th century. All subregions exhibit distinct century-scale climate changes: an extension of the vegetation period by approximately 70–80 days; a summer warming rate of around 4 °C; and 20–30 mm more summer rainfall interrupted by a greater number of drought period days. It can be expected that present-day climate analogues for these future climate types are spatially far away from their current location in Franconia.

**Table 1:** Future climatic characteristics of the nine clusters identified across the study domain derived at the end of the 21st century and their mean changes relative to the 1970–1999 reference period.

	Geographical description	Height a.s.l.^27^	Mean values 2070–99 (difference regarding 1970–99)			
			Temperature [°C] (April through September)	Precipitation [mm] (April through September)	Drought period days (April through September)	Length of vegetation period [days]
1	Highest ridges of low mountain regions	~ 800 to > 1000 m	16.1 (+ 4.0)	494.9 (+ 28.0)	32.0 (+ 7.6)	284.8 (+ 79.5)
2	Low mountain regions	~ 550 to 700 m	17.0 (+ 3.9)	451.1 (+ 19.7)	38.8 (+ 8.9)	304.8 (+ 76.0)
3	Higher plateaus	~ 500 to > 600 m	17.0 (+ 4.0)	458.8 (+ 29.9)	35.7 (+ 7.2)	300.7 (+ 79.5)
4	Foothills of low mountain regions	~ 300 to 500 m	17.6 (+ 3.9)	412.7 (+ 28.8)	40.6 (+ 7.9)	312.4 (+ 75.7)
5	Hill ridge regions	~ 400 to 550 m	17.7 (+ 4.0)	414.2 (+ 25.0)	38.4 (+ 7.1)	310.0 (+ 76.2)
6	Middle Franconian plateaus	~ 300 to 400 m	18.1 (+ 4.1)	410.1 (+ 25.1)	37.1 (+ 6.9)	310.5 (+ 74.3)
7	Transition zone	~ 250 to 350 m	17.9 (+ 3.9)	378.9 (+ 27.1)	42.2 (+ 7.3)	316.1 (+ 74.1)
8	Downstream main valley	~ 100 to 175 m	18.2 (+ 3.8)	367.7 (+ 28.1)	43.9 (+ 8.2)	322.9 (+ 70.0)
9	Middle reaches of the main valley and Reg(d)nitz valleys	~ 175 to 300 m	18.4 (+ 3.9)	362.5 (+ 27.7)	42.9 (+ 6.9)	318.6 (+ 71.9)

### Climate analogue regions under present-day and future conditions

Climate analogue regions are identified for each of the nine Franconian subregions individually. Each cluster is characterized by a range of values of the leading two PCs stretching over the grid boxes attributed to a given cluster. The upper and lower limits of the ranges for the entire study region are used to demarcate possible analogue regions. This approach guarantees that climate analogues exhibit a climate within the same margins as the overall target region. For further subdivision regarding the Franconian clusters, a standardized Euclidian distance measure is used, applying a weighting scheme according to the portion of variance accounted for by each PC. In addition to the regional climate, soil characteristics are crucial for crop growing. Therefore, only analogue regions with matching soil properties in terms of soil texture and pH value are admitted (see Methods). The pattern of the nine clusters remains identical for subsequent analyses. The E-OBS observational dataset that is considered to describe present-day climatic conditions across Europe, is also transformed into the PC space derived from the future Franconian climate to create a common variable space based on the same 28 climate indicators. Although analogue regions could be identified worldwide, our study is restricted to Europe due to the quality and resolution of the analysed data, but also to safeguard a certain comparability with respect to agricultural practice and knowhow.

In the first step, the climate analogue regions are determined for the current climate of Franconia for two purposes: (1) to address the motivation of this case study assuming that the heterogeneity of Franconian climate is representative for large parts of Central European climate, and (2) to visualize the spatial shift of the climate analogues until the end of our century. This analysis is entirely based on the E-OBS dataset over the 1993–2022 period (except for the delineation of Franconian subregions). The present-day climate analogue regions are displayed in the top panel of Fig. 2. The target region of Franconia is highlighted with a bold frame and, of course, is an analogue of its own present-day climate when considering the region as a whole (or subregions based on recent observational data as depicted in Supplementary Fig. S3. This clustering leads to some differences but an overall similar pattern. However, the comparability with regards to the analogue regions of future Franconian climate would go astray when using differing Franconian subregions). Additional climate analogues stretch from higher elevation areas in the Balkan region to the flat part of southern Sweden, giving support for the conjecture that our study domain is representative of Central European climate, excluding the maritime west, the continental east and most of the Mediterranean south. Colder and wetter sub-climates in the Franconian low mountain areas (dark blue) resemble the climate of southern Sweden. Cold to temperate sub-climates (light blue) prevail in southern Bavaria and along the ridge of the Dinaric Alps. Temperate to warm climate types (yellow) can also be found in central and northern Germany. Warmer and drier sub-climates (red) are nowadays found in eastern Germany and in the Serbian basin. There are also some disperse grid boxes identified in higher-elevation areas of southern France and northern Spain.

Until the end of the 21st century—based on a multi-model mean of seven distribution-corrected CORDEX simulations—enhanced radiative forcing imposes a distinct shift of the climate analogue regions towards the southwest, the south and low-elevation areas around the northern Mediterranean Sea (bottom panel of Fig. 2). Widespread analogues are revealed for the cooler Franconian subregions (blue colours), covering a belt-like structure from the Baltic Sea to the Massif Central in France. Only for subregion 1, future analogues can be found in Franconia itself, namely in the northwestern parts. These future sub-climate types are also found at higher altitudes of mountainous regions in southern Europe: e.g., Pindus in Albania/Greece, Dinaric Alps, Apennine Mountains, Alpine fringe, Pyrenees, and mountain ranges in northern Spain. For instance, today’s sub-mountainous climate of eastern Franconia with maximum altitudes beyond 1,000 m asl. will then experience the climate of lowland northeastern Germany with hot and dry summers.

The relatively warm and dry subregions of Franconia (red colours) find their climate analogues further southward and at strictly lower elevations. At first sight, the pattern appears more disperse stretching from Galicia in northern Spain to northern Greece. Yet, a common feature is that these grid boxes are located along the southernmost rims of southern European mountain ranges like the Pyrenees in Spain, the Alps and the Apennine Mountains in Italy and the Dinaric Alps in the Balkan region. In addition, this future sub-climate type nowadays occurs in the Gascony Plain in southwestern France and the Padan Plain in northern Italy. There is another interesting detail: the climate analogues in the top panel of Fig. 2 reveal that the warm Franconian subregions are already represented in the Balkan region under present-day conditions. Until the end of the 21st century, they exhibit a shift from higher elevations at the leeward side of the Dinaric Alps to much lower elevations at the coastal side. In consideration of the typical landscapes in these analogue regions it is obvious that future land use pattern in Franconia may change substantially.

### Changes in prevalent crops derived from the analogue regions

**Fig. 3:**
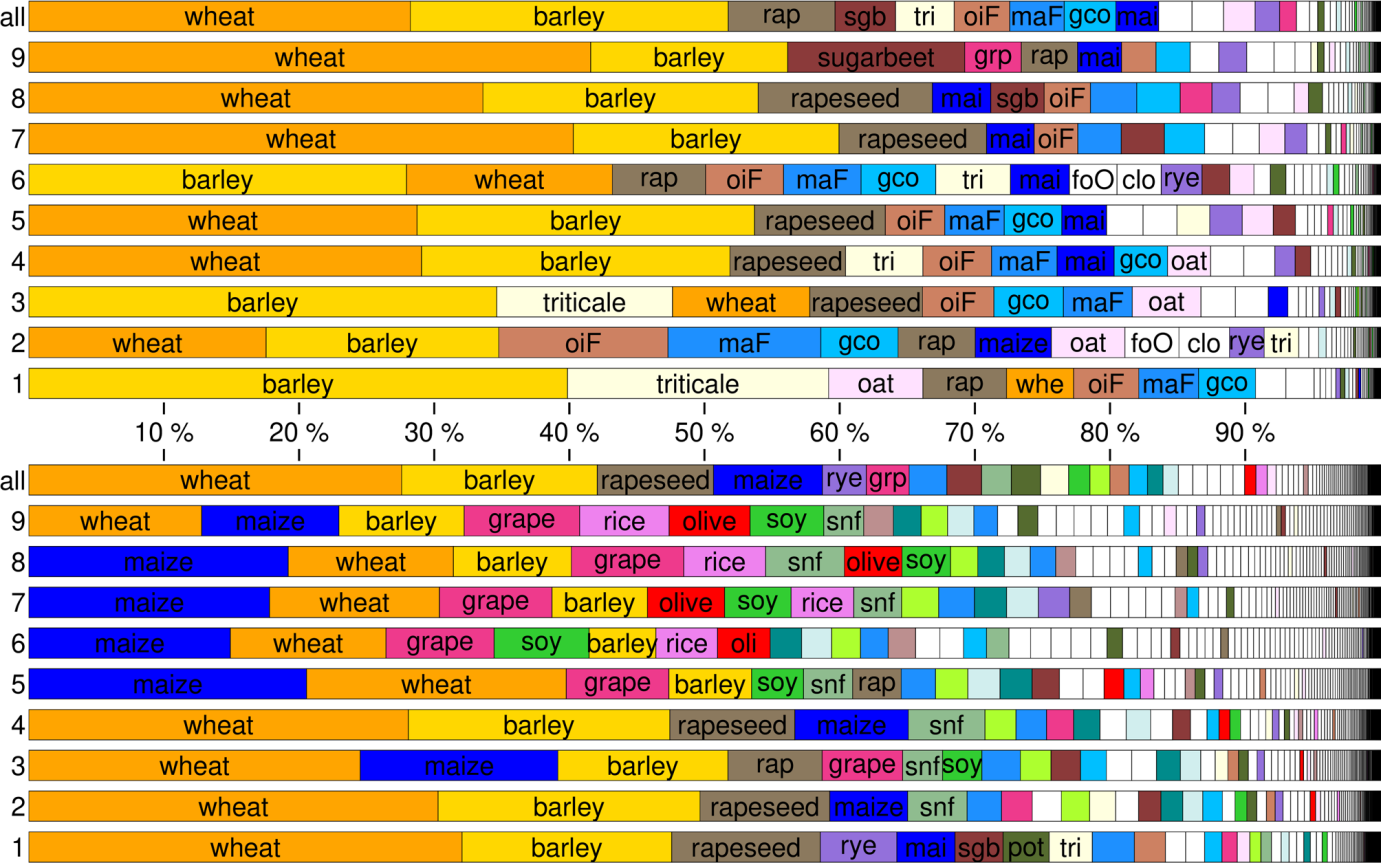
Most prevalent crops in the nine Franconian subregions and in Franconia as a whole under present-day conditions (top) and as derived from the analogue regions indicating climate conditions in Franconia at the end of our century (bottom). A list of all crop names and abbreviations can be found in Supplementary Table S3. Figure created using GMT - The Generic Mapping Tools, Version 6.5.0 (https://www.generic-mapping-tools.org/).

Instead of drawing a qualitative conclusion about climate-induced future land use in Franconia, we pursue a quantitative investigation based on high-resolution land cover data that are area-wide available for the present-day target region as well as all identified climate analogue regions. The lately published CROPGRIDS dataset^25^ (see Methods for details) allows for assessing changes in crop spectra. The present-day crop spectrum of entire Franconia as well as the nine subregions is illustrated in the top panel of Fig. 3: wheat (28% of cropped area) and barley (24%) are the most common crops in Franconia, followed by rapeseed (8%) as well as sugar beet, triticale, forage oilseed, forage maize and green corn (4% each). While the prevalence of wheat (28%) and rapeseed (9%) cropping barely changes in the future (Fig. 3, bottom), the cultivation of barley will decrease considerably (14%). Fodder crops (forage maize, forage oilseeds, others) and sugar beet also lose, while maize (8%), rye and grapevine (3% each) gain importance.

When taking a closer look at individual subregions and their corresponding climate analogues, the changes become even more striking. In the future, maize will be the prevalent crop in analogue regions 5–8 (up to 21% of cropped area in region 5). In regions 3 and 9, it holds the second rank. Especially grapes become more prominent in most subregions (rank 3–5, max. 9% in region 9) while, under present-day conditions, they make only a minor contribution to the two warmest subregions in Franconia. Two future crops becoming important are unprecedented in Franconia: rice (max. 7% in region 9) and olives (max. 6% in region 9). Among the crops that are nowadays not grown in Franconia but will only cover smaller areas in the future, the following have to be mentioned: almonds (max. 2.2% in region 9), forage rye (max. 1.9% in region 4), oranges, hazelnuts, peaches, tangerines, chestnuts and pumpkins (max. 0.3–1.5% listed in declining order, all in region 6), and sorghum (max. 0.7% in region 4). Supplementary Figs. S4 and S5 focus on these minor crops and their percentage of area in region 4 and 6, respectively. From the perspective of today’s agriculture in Germany, these crops are particularly exotic and may imply the largest investments in the transformation process of Franconian agriculture.

## Discussion and conclusion

This study sheds light on the future crop spectra that may be cultivated in the region of Franconia, southern Germany, when undamped climate change continues until the end of our century. Some water-demanding and less heat-resistant crops (e.g., barley and sugar beet) will presumably lose importance, whereas other already existing crops might become more prevalent (e.g., wheat, maize and rapeseed). Even more striking is the anticipated emergence of new crops that are nowadays typical for Mediterranean growing areas (e.g., olives, rice and even citrus fruits).

One of these new crops requiring a critical review is rice. Our analysis predicts improved growing conditions for rice in the warm and relatively dry Franconian subregions 6–9. However, rice is a crop with high water requirements^29^ and, therefore, mostly irrigated^30^, e.g., in the Po Valley in northern Italy^31,32^ where water abundance originating from Alpine snow reservoirs is generally high^33,34^. This situation is not given in Franconia. Therefore, a high prevalence of rice cropping is not to be expected here for hydrological constraints although the climatic boundary conditions may become appropriate.

In contrast to this, the cultivation of Mediterranean olive trees usually happens without irrigation^35^. Nevertheless, if annual precipitation falls below 350 mm, prosperity is inhibited and high yields are only to be expected from 500 mm onward^36^. In fact, a northward shift of Italian olive cropping was detected, partly into regions with higher elevation, as a consequence of warmer conditions in recent decades^37,38^. Olive cultivation is expected to move further towards north in the future, possibly vanquishing the Alpine barrier, while growing conditions in many of today’s cropping areas may deteriorate mainly due to water stress^39–42^. Yet, water shortage in summer could also become a limiting factor in future Franconian climate. Not explicitly included in our analysis are crop-specific chilling demands and tolerance to frost. Olive trees possess specific chilling requirements which depend on the variety cultivated^40,43^. Harming effects for olives are expected for temperatures below − 8.3 °C^36^ which may very rarely be undercut in future Franconian winters.

Altogether, we have tried to optimize the identification of analogue regions based on natural landscape characteristics such as multivariate regional climate and soil properties. By explicitly including the knowledge of stakeholders in the selection of climate indices, the overly theoretical scientific point of view was extended to ensure the practical significance of the study. However, regarding the adoption of new crops, numerous barriers might exist. Studies on the implementation of changes in agriculture mention, e.g., regulations and policy support; availability of seeds, infrastructure, logistics, and markets; costs and profitability. Behavioural factors on the farmers’ side include perception of costs, benefits, control, and risks; information and knowledge; social and cultural factors as well as general objectives and motivation of farmers^44–46^. Furthermore, many additional factors play a role, some of them may serve as tools of adaptation, e.g., new cultivation techniques and irrigation. They may reduce the sensitivity of crop growing to climate change and mitigate or delay the transformation in the agricultural sector. Another limitation of this study pertains to disregarded boundary conditions such as day length, radiation angle, exposition and soil hydrology. Although two of the 28 utilized indices encompass latitudinal information and these are highly represented by one of the applied PCs each, photoperiodicity is not explicitly included in the study. Nevertheless, breeding of crops has resulted in cultivars with different features of day-length sensitivity allowing for a more latitudinally-independent cultivation [e.g.^47–49^]. Additionally, the horizontal resolution of the considered datasets is state-of-the-art but still does not meet the micro-climatic field scale of agricultural production.

To sum up, although the comprehensiveness of the spanned space of climate features is inevitably incomplete and actual feasibility of crops might be limited by various factors despite climatic suitability, our analysis has quantitatively assessed potential future crop spectra and foreshadows a radical transformation process for Central European agriculture.

## Methods

### Selection of climate indicators for characterizing the climate of Franconia

In order to characterize the climate of the study in an agriculturally relevant way, a list of indicators has been collected from stakeholders (farmers, representatives of local authorities) collaborating in the EU-funded project BigData@Geo 2.0. This has been done by means of personal conversations – during workshops and farm visitations – as well as based on a survey sheet (Open question: “Which climatic parameters, processes, events, or variables do you consider important for your crops?”, see also Supplementary Table S1 as well as supplementary material of ref^10^). spread via e-mail. With this, our research has involved human participants. We confirm that all this research has been performed in accordance with relevant guidelines and regulations. We have consulted with the Medical Ethics Committee at Julius-Maximilians-University of Würzburg, which has waived ethical approval. As the involvement of human participants has not included any ethical parameters nor medical experiments, the obtaining of informed consent from the involved subjects has not been applicable. Regarding the rights of use of the collected data, we declare that all participating respondents are involved in the BigData@Geo 2.0 project and have signed a cooperation agreement, thereby agreeing to participate in the project and allowing the researchers to use and disseminate the collected data. The completion and refinement of the list has been conducted based on literature as well as knowledge of the involved researchers.

### Internal segmentation of future Franconian climate

The 28 climate indicators resulting from the selection process described in the previous paragraph are calculated based on seven regional climate model (RCM) simulations from the EURO-CORDEX initiative^50,51^ driven by various global climate models (see Supplementary Table S3) using the RCP 8.5 scenario^52^. The 0.11° resolution daily data is nearest neighbour-interpolated onto the 0.1° grid of E-OBS gridded observations (see section below) and bias-corrected by means of empirical quantile mapping with 101 quantiles and linear interpolation [cf.^53–55^] before calculating the indices. Finally, the quality-controlled indicators are averaged over the 2070–2099 period and over all seven RCM ensemble members. By using this multi-model mean of seven distribution-corrected simulations, the variability of climate model simulations is accounted for and a fitting of the model data regarding properties of the observational data is obtained. Although this use of climate model ensembles is a well-established approach, one always has to keep in mind that it’s in the nature of models to be deficient. Uncertainties arise from various sources like insufficient knowledge of the climate system, necessary simplifications, forcing uncertitudes as well as internal variability and RCM downscaling techniques [e.g.^50,55^].

Many of the resulting spatial index patterns are highly correlated, with Pearson correlation coefficients ranging from *r* = 0.9956 (between heat days and days in heatwaves) to *r* = 0.45 (minimum daily temperature amplitude per year with heat days). To account for the high intercorrelation and the associated redundancy of information among the index data, a principal component analysis (PCA) [e.g.^56–58^] is conducted, using a correlation matrix. In a PCA, independent (orthogonal) principal components (PCs) are deduced from intercorrelated data by rotational transformation, sorted regarding their share of variability. By doing so, firstly, dominant patterns are condensed, and secondly, the dimensionality is reduced, meaning that most of the overall variance is represented by a small number of variables. The resulting first two principal components (PC1 and PC2) represent 62.0% and 17.4% of the overall variance, respectively, meaning that almost 80% of the entire variability is encompassed by them. As all indices are represented by one of the two (with at least |r|=0.70, except for the minimum daily temperature amplitude per year with max. *r* = 0.35, see Supplementary Fig. S2), PC1 and PC2 are used for further analysis. The indices that are most reflected in the principal components (PCs) are the Huglin index (PC1, *r* = 0.98) and the annual temperature amplitude between maximum and minimum temperatures (PC2, *r* = 0.92). PC1 generally represents temperature, which corresponds highly with precipitation due to topographic features. PC2 generally represents continentality and seasonality. The loadings (empirical orthogonal functions, EOFs) and PCs are shown in Supplementary Fig. S6.

For the assessment of subregional climate types, a cluster analysis is carried out, weighting PC1 and PC2 according to their portion of explained variance. First, a hierarchical cluster analysis is performed, prescribing a cluster number between 2 and 10. Each analysis is evaluated by means of the average Euclidian distance among all grid cells. This is followed by a k-means re-clustering of the results in the same manner. Afterwards, the variability inside the clusters is compared to the one between the clusters. The minimum of this ratio defines the best number of clusters which is 9 in the case on hand.

### Search for analogue regions in today’s observed climate across Europe

The recent climate state is depicted by the E-OBS observational data set in version 28.0^59,60^, which forms the basis for the calculation of 1993 to 2022 average index values for the whole European domain. The data matrix is then projected onto the EOFs of the future climate as derived from the RCM multi-model ensemble. The attribution to the future climate in Franconia is guided by the lower and upper limits of the PC scores in the whole region. This proceeding ensures that climate analogues exhibit the same climate characteristics as the Franconian study region. Within these margins, the attribution to the sub-regions is based on an Euclidian distance to enable a weighting between the first and second PC, accounting for different portions of total variance.

### Inclusion of soil characteristics

As growing conditions are not solely determined by climate constraints, only grid cells with fitting soil features are approved. A matching is anticipated in the case of complying classes of clay content, sand content, and soil pH values. Based on data of the Harmonized World Soil Database^61^, the texture-related classes are divided at 33.33% and 66.67%, the pH value (pH H_2_O integrated over the upper 100 cm) from the Soilgrids Dataset^62^ is split up at values of 6 and 8.

### Crop cultivation in the respective regions

Once the nine subregions in Franconia and corresponding analogue regions identified, the lately published CROPGRIDS dataset^25^—a global dataset regarding the areal coverage of 173 crops in 0.05° resolution for the year 2020 based on a dataset for around 2000^63^, 27 other published datasets, subnational data of 52 countries as well as national-level statistics of FAOSTAT for 2020—is consulted to assess the current and potential future crop cultivation in Franconia and its subregions. For this, the cultivated area of each grid cell of interest is analysed with respect to the area share of each featured crop. A list of all possible crops is given in Supplementary Table S4.

## Supplementary Information

Below is the link to the electronic supplementary material.

Supplementary Material 1

## Data Availability

The E-OBS data is available via the Copernicus Climate Change Service (https://cds.climate.copernicus.eu/datasets/insitu-gridded-observations-europe?tab=overview). The CORDEX data can be accessed via the Earth System Grid Federation data nodes (e.g., http://esgf-data.dkrz.de/). The CROPGRIDS data can be downloaded from a figshare repository (10.6084/m9.figshare.22491997). The CORINE land cover data is provided by the Copernicus Land Monitoring Service (https://land.copernicus.eu/en/products/corine-land-cover/clc2018).

## Code availability

The code to replicate all findings will be provided upon request by the corresponding author.
