# Supplementary material for: Undamped climate change poses the need for substantial shifts in cultivated crop types in Germany
Source: Sci Rep. 2026 Mar 2;16:7945. doi: 10.1038/s41598-026-42040-x (PMC12957499; doi:10.1038/s41598-026-42040-x)
Supplement: Supplementary file 1 — Supplementary Material 1 [file 41598_2026_42040_MOESM1_ESM.docx]

**Supplementary Information**


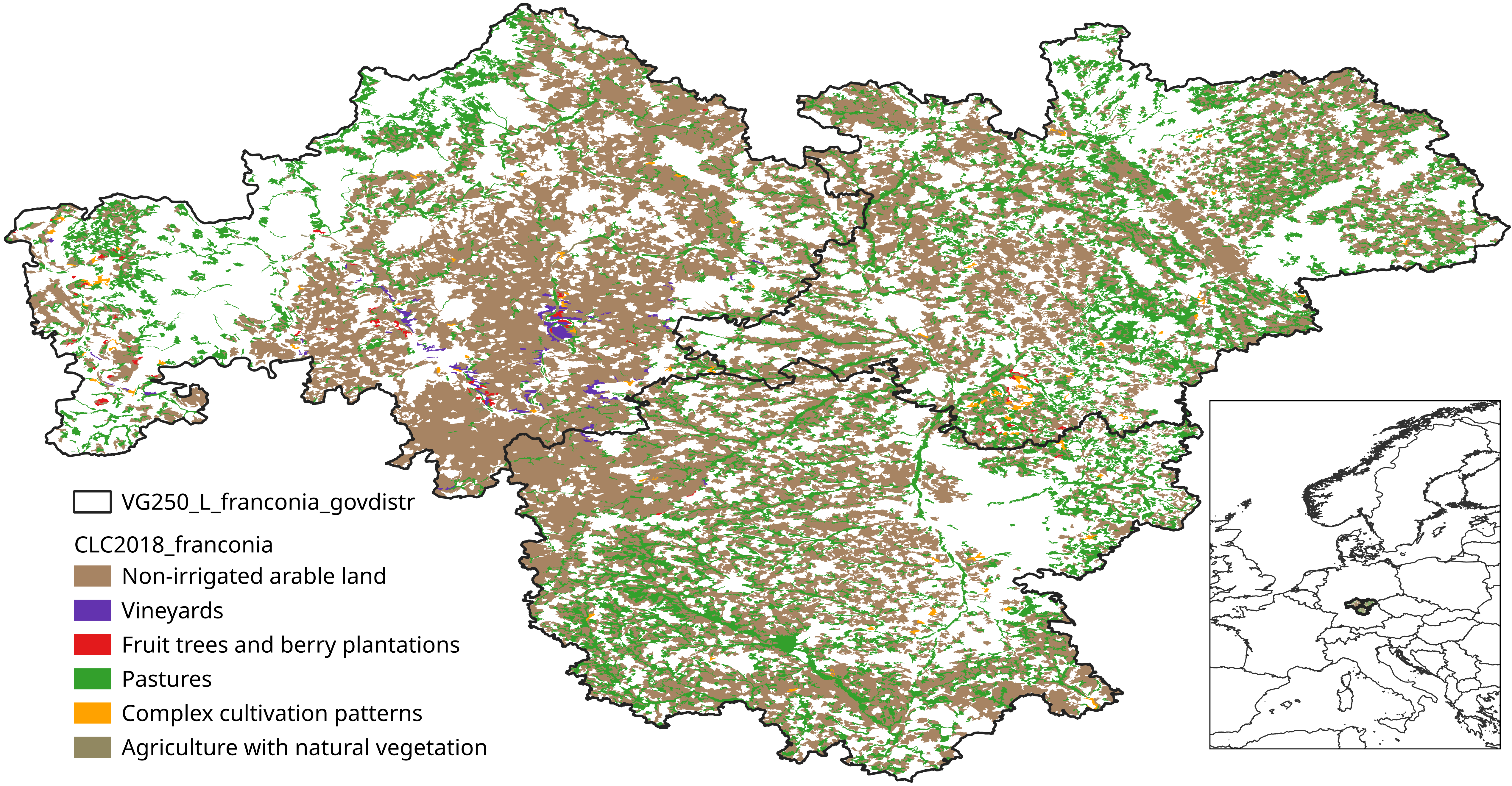
*Supplementary Figure S1. The three governmental districts of Franconia (Lower Franconia in the northwest, Middle Franconia in the south, Upper Franconia in the northeast) [S1] and agricultural land cover [S2]. The small figure on the lower right shows the position in Europe with country data from EuroGeographics and UN-FAO [S3]. Map created using QGIS Version 3.42 (https://qgis.org/).*


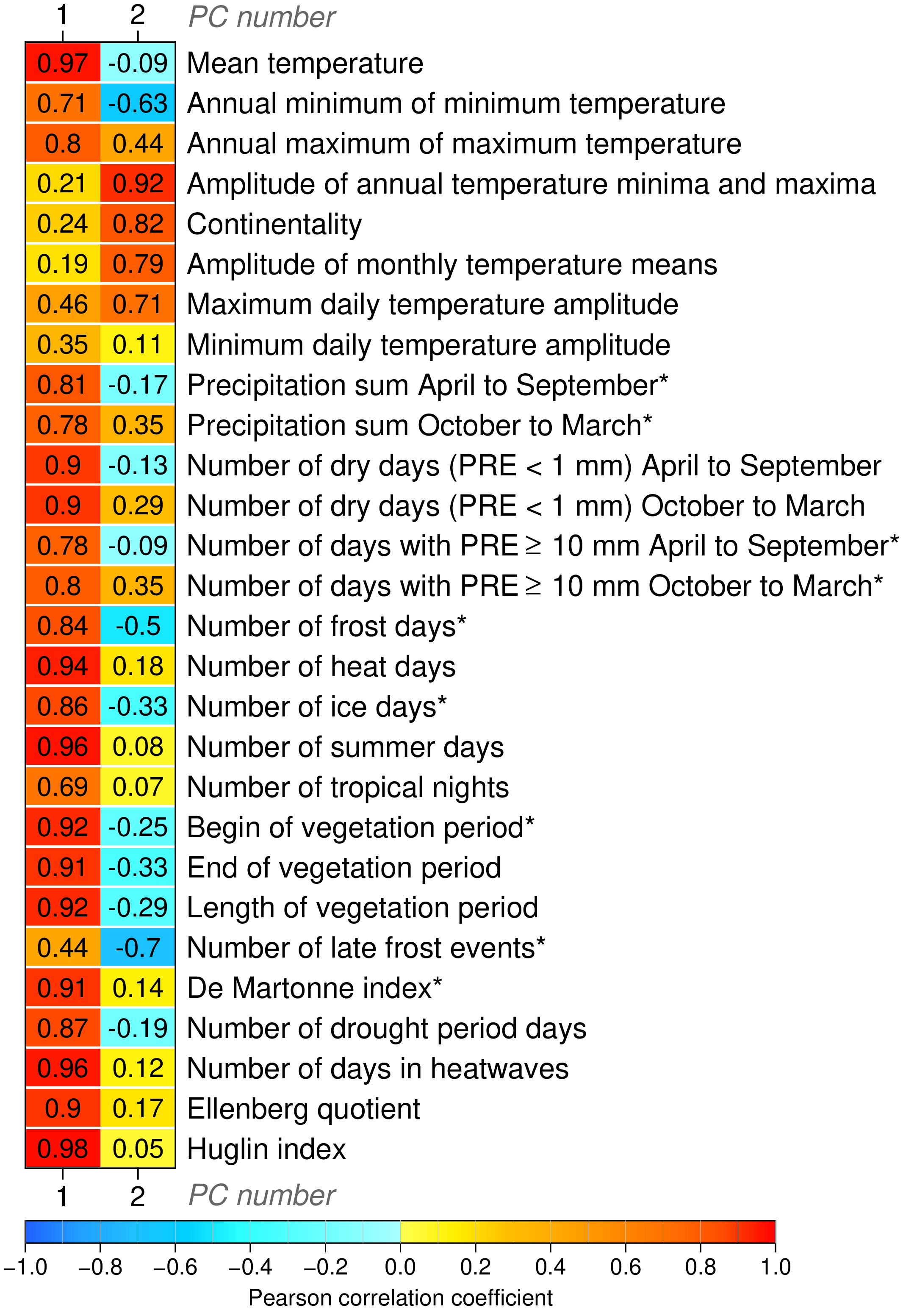


*Supplementary Figure S2: Original climate indices included in the study and their Pearson correlation coefficient with the first (explained variance 62.0 %) and second (explained variance 17.4 %) principal component. Figure created using GMT - The Generic Mapping Tools, Version 6.5.0 (https://www.generic-mapping-tools.org/).*


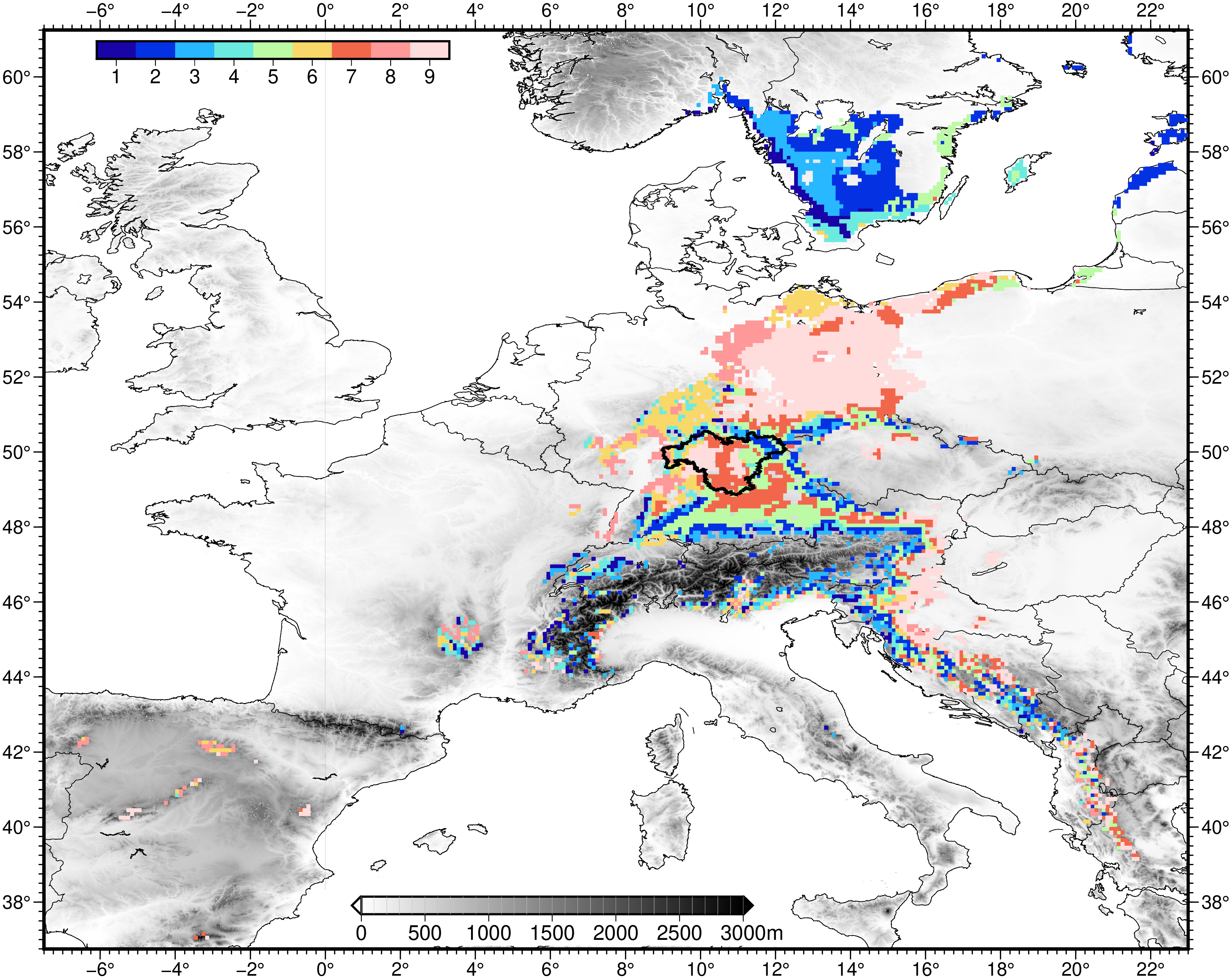
*Supplementary Figure S3: Analogue climates of Franconia under present-day (1993–2022) conditions using an internal segmentation of Franconia based on present-day climate. Franconia is framed. As clustering and search for analogues is entirely based on the same data, subregions inside Franconia coincide with the associated analogue regions inside Franconia. Clusters differ from those in Table 1 and Figure 1 of the main manuscript, where clustering refers to future (2070–2099) climate projections. Thus, a different colouring was employed. Figure created using GMT - The Generic Mapping Tools, Version 6.5.0 (https://www.generic-mapping-tools.org/). Relief shading based on SRTM15 data [S4]; shorelines from GSHHG [S5].*


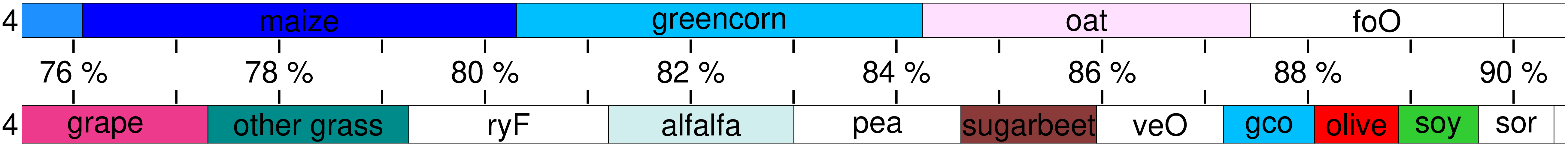
*Supplementary Figure S4: Present-day (top) and future (bottom) crops grown in subregion 4, focussing on minor crops in the 75.5 to 90.5 % range of total crop cover. Figure created using GMT - The Generic Mapping Tools, Version 6.5.0 (https://www.generic-mapping-tools.org/).*


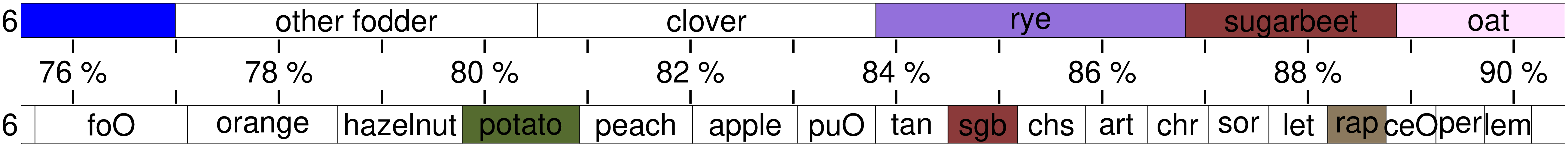
*Supplementary Figure S5: Present-day (top) and future (bottom) crops grown in subregion 6, focussing on minor crops in the 75.5 to 90.5 % range of total crop cover. Figure created using GMT - The Generic Mapping Tools, Version 6.5.0 (https://www.generic-mapping-tools.org/).*


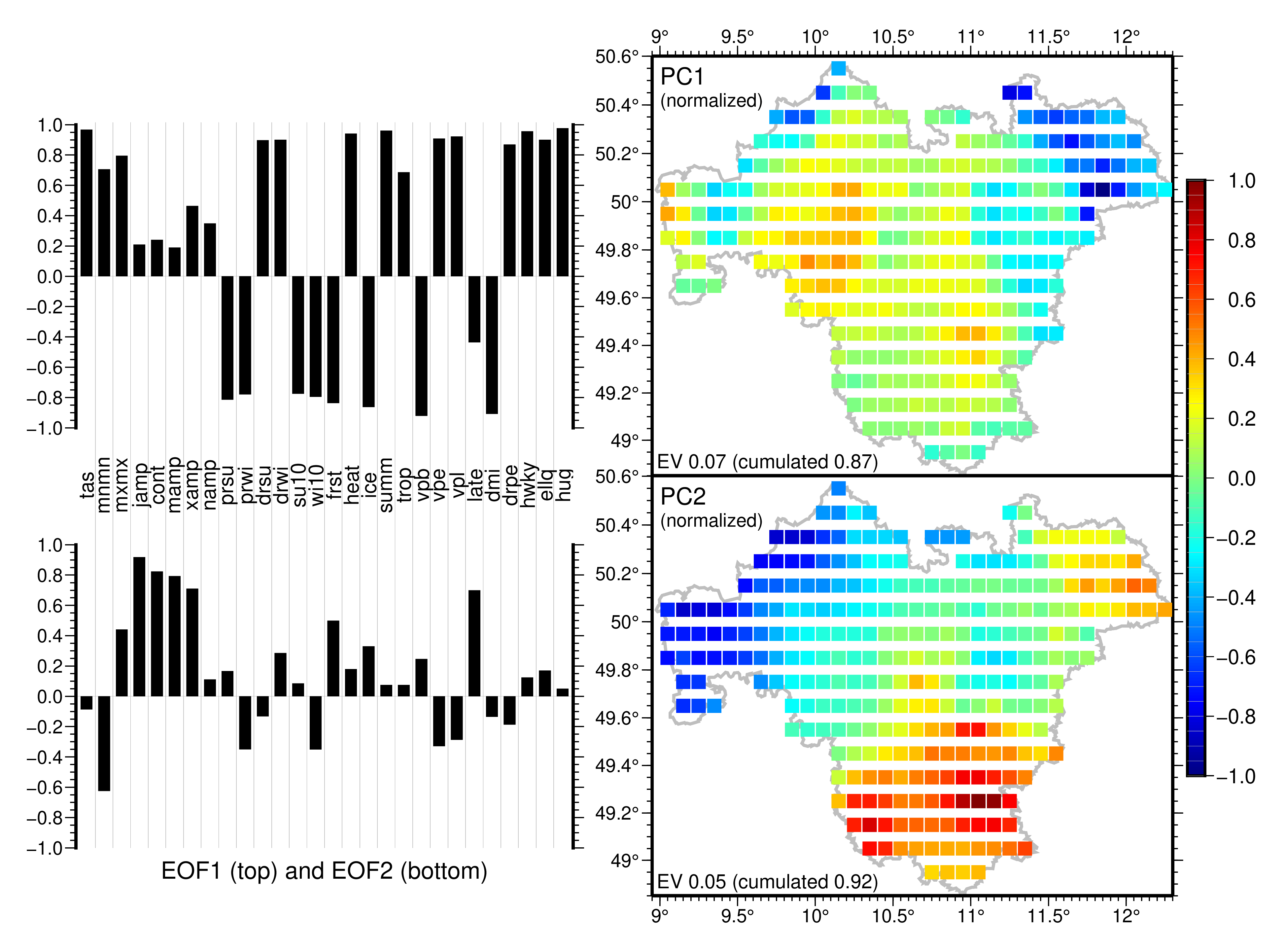
*Supplementary Figure S6: Loadings (left) and scores (right) of the first two principal components (PCs) based on 28 temperature- and precipitation-related indicators of Franconian climate at the end of the 21^st^ century. The first PC accounts for 62 % of the overall variance and reflects temperature as well as precipitation features (see loadings on the left) as guided by the topography of the region (see scores on the right, cf. Figure 1). The second PC accounts for 17 % of the overall variance and reflects continentality and seasonality. Figure created using GMT - The Generic Mapping Tools, Version 6.5.0 (https://www.generic-mapping-tools.org/).*

*Supplementary Table S1: Survey among Franconian farmers and inference of indices. Answers to the question “Which climatic parameters, processes, events, or variables do you consider important for your crops?” surveying more than 30 farmers (of which 24 replied) and indices the parameters were translated to (see Supplementary Table S2). Other parameters were named 3 times at maximum and/or not inferable from minimum, mean, maximum temperature or precipitation.*

| **Stated parameters** | **Count** | **Translated into indices** |
| --- | --- | --- |
| Precipitation (generally, distribution, timing / seasonality / annual cycle, frequency) | 21 | prsu, prwi,drsu, drwi, su10, wi10 |
| Dryness | 17 | drsu, drwi, dmi, drpe |
| Temperature (generally, distribution) | 16 | tas, mnmn, mxmx, aamp, mamp, xamp, namp |
| Late frost | 16 | late |
| Frost | 12 | mnmn, frst, ice |
| Heat (incl. summer and heat days) | 11 | mxmx, heat, summ, trop, hwky |
| Vegetation period | 9 | vpb, vpe, vpl |
| Heavy precipitation | 8 | su10, wi10 |

*Supplementary Table S2: Climate indices used in this study resulting from a survey among farmers and extended by vegetation-related indicators. Used variables: temperature T, precipitation PRE, latitude LAT*

| **Abbr.** | **Climate indicator** | **Definition / description** |
| --- | --- | --- |
| Temperature mean, extremes, and amplitudes | | |
| tas | Mean temperature | |
| mnmn | Annual minimum of minimum temperature | |
| mxmx | Annual maximum of maximum temperature | |
| aamp | Amplitude of annual temperature minima and maxima | |
| cont | Continentality | $cont=aamp\frac{1.7}{sin\left( LAT \right)}-20.4$ [S6]  This index serves to distinguish more maritime from more continental climate types:  0 ≤ cont ≤ 33: maritime  34 ≤ cont ≤ 66: continental  67 ≤ cont ≤ 100: extreme continental |
| mamp | Amplitude of monthly temperature means | |
| xamp | Maximum daily temperature amplitude | |
| namp | Minimum daily temperature amplitude | |
| Half-year precipitation parameters | | |
| prsu | Precipitation sum April to September | |
| prwi | Precipitation sum October to March | |
| drsu | Number of dry days (PRE < 1 mm) April to September | |
| drwi | Number of dry days (PRE < 1 mm) October to March | |
| su10 | Number of days with PRE ≥ 10 mm April to September r | |
| wi10 | Number of days with PRE ≥ 10 mm October to March | |
| Threshold days | | |
| frst | Number of frost days | Days with T_min_ < 0 °C |
| heat | Number of heat days | Days with T_max_ ≥ 30 °C |
| ice | Number of ice days | Days with T_max_ < 0 |
| summ | Number of summer days | Days with T_max_ ≥ 25 °C |
| trop | Number of tropical nights | Days with T_min_ ≥ 20 °C |
| Vegetation period and late frost | | |
| vpb | Begin of vegetation period | First time in year with ≥ 5 consecutive days with T_mean_ ≥ 5° C |
| vpe | End of vegetation period | First time in year with ≥ 5 consecutive days with T_mean_ < 5° C |
| vpl | Length of vegetation period | vpe – vpb |
| late | Number of late frost events | Number of days with T_min_ < 0 after begin of vegetation period (until June 30^th^) |
| Drought and heat indices | | |
| dmi | De Martonne index | $dmi=\frac{{PRE}_{annual}}{10+T_{annualmean}}$ [S7,S8,S9]  This index is a measure of dryness. Classes are:  0 ≤ dmi < 10: no (regular) runoff  10 ≤ dmi < 20: temporary runoff  20 ≤ dmi < 30: intermediate  dmi > 30: humid |
| drpe | Number of drought period days | Drought period: at least 10 days PRE < 1 mm |
| hwky | Number of days in heatwaves | A heatwave is defined as [S10]:  ≥ 3 days with T_max_ ≥ 30 °C and  mean T_max_ ≥ 30 °C over the period and  T_max_ ≥ 25 °C over the period |
| Vegetation-related indices | | |
| ellq | Ellenberg quotient | $ellq=1000\frac{T_{mean,July}}{{PRE}_{annual}}$ [S11]  This index describes the type of natural forest climate. Historically, Franconia encompasses all of the three main classes:  < 20 cool/wet (beech dominating)  20–30 transition (beech forests with oaks)  > 30 warm/dry (mixed oak, mostly no beech) |
| hug | Huglin index | $hug=k\sum\frac{\left( T_{mean}-10 \right)+\left( T_{max}-10 \right)}{2}$ [S12]  latitudinal-dependent factor k: [S13]  for latitudes ≥ 50°: $k=1.06$  for latitudes < 50°: $k=1.02+0.04\cdot\frac{LAT-40}{10}$  This index describes the thermal wine-growing suitability of a given region. For hug < 1500, wine growing is not recommended, rising values correspond to conditions favouring more thermophilous cultivars. |

*Supplementary Table S3: The available seven very high-resolution regional climate model projections under RCP8.5 emission scenario from the CORDEX initiative, along with their driving Global Climate Model (GCM) and responsible research institution.*

| **Institution** | **RCM** | **GCM** |
| --- | --- | --- |
| Climate Limited-Area Modelling Community | CCLM4-8-17 | MIROC5 |
| Centre National des Recherches Météorologiques France | ALADIN63 | CNRM-CM5 |
| Danish Meteorological Institute | HIRHAM5 | NorESM1-M |
| Helmholtz-Zentrum Geesthacht, Climate Service Center Germany | REMO2015 | MPI-ESM-LR |
| Institute Pierre-Simon Laplace | WRF381P | HadGEM2-ES |
| Royal Netherlands Meteorological Institute | RACMO22E | EC-EARTH |
| Swedish Meteorological and Hydrological Institute, Rossby Centre | RCA4 | IPSL-CM5A-MR |

*Supplementary Table S4: List of crops occurring in the target region and various climate analogue regions with their abbreviations used in the figures (in brackets), according to ref. [S14 [=25 in main manuscript]].*

| alfalfa (alf)  almond (alm)  apple  artichoke (art)  barley  cherry (chr)  chestnut (chs)  clover (clo)  fodder maize (maF)  fodder oilseed (oiF)  grape (grp)  greencorn (gco)  hazelnut  lemon & lime  lettuce (let)  maize (mai)  mixedgrass (mgr)  oat  olive (oli)  orange | peach & nectarine  pea  pear (per)  potato (pot)  other cereal (ceO)  other fodder (foO)  other grass (grO)  other pulse (puO)  other vegetable (veO)  rapeseed (rap)  rice  fodder rye (ryF)  rye  sorghum (sor)  soybean (soy)  sugarbeet (sgb)  sunflower (snf)  tangerine, clementine, mandarin, satsuma (tan)  triticale (tri)  wheat (whe) |
| --- | --- |

**Supplementary References**

S1 Federal Agency for Cartography and Geodesy & GeoBasis-DE. Administrative areas 1 : 250 000. VG250. Shape data set. (2017).

S2 European Union's Copernicus Land Monitoring Service: CORINE land cover 2018. <https://doi.org/10.2909/71c95a07-e296-44fc-b22b-415f42acfdf0> (2020).

S3 EUROSTAT. Countries, 2020 - Administrative Units. Shape data set. (2020).

S4 Tozer, B. et al. Global bathymetry and topography at 15 src sec: SRTM15+. *Earth and Space Science* **6(10),** 1847–1864. <https://doi.org/10.1029/2019EA000658> (2019).

S5 Wessel, P. & Smith, W. H. F. A global, self-consistent, hierarchical, high-resolution shoreline database. *Journal of Geophysical Research: Solid Earth* **101(B4),** 8741–8743. <https://doi.org/10.1029/96JB00104> (1996).

S6 Gorczynski, L. The calculation of the degree of continentality. *Monthly Weather Review* **50(7),** 370. <https://doi.org/10.1175/1520-0493(1922)50%3C370b:TCOTDO%3E2.0.CO;2> (1922).

S7 de Martonne, E. Aerisme, et índices d’aridite. *Comptes Rendus de L’Academie Des Sciences* **182,** 1395-1398 (1926).

S8 de Martonne, E. L’indice d’aridité. *Bulletin de l’Association de Géographes Français* **9,** 3–5 (1926).

S9 de Martonne, E. Nouvelle carte mondial de l’indice d’aridité. *Annales de Géographie* **51(288),** 241–250 (1942).

S10 Kyselý, J., Kalvova, J. & Květoň, V. Heat waves in the south Moravian region during the period 1961-1995. *Studia Geophysica et Geodaetica* **44(1),** 57–72. <https://doi.org/10.1023/A:1022009924435> (2000).

S11 Leuschner, C. & Ellenberg, H. *Ecology of Central European Forests. Vegetation Ecology of Central Europe Volume I*. <https://doi.org/10.1007/978-3-319-43042-3> (Springer, 2017).

S12 Huglin, M. P. Nouveau mode d’évaluation des possibilités héliothermiques d’un milieu viticole. *Comptes Rendus de l’Académie d’Agriculture de France* **64,** 1117–1126 (1976).

S13 Stock, M. et al. *Perspektiven der Klimaänderung bis 2050 für den Weinbau in Deutschland (Klima 2050). Schlußbericht zum FDW-Vorhaben Klima 2050.* PIK Report 106 (Potsdam Institute for Climate Impact Research, 2007).

S14 Tang, F. H. M. et al. CROPGRIDS: a global geo-referenced dataset of 173 crops. *Scientific Data* **11(1),** 1–14. <https://doi.org/10.1038/s41597-024-03247-7> (2024).
